# Aerobic biodegradation of untreated polyester–polyether urethanes by newly isolated yeast strains *Exophilia* sp. NS-7 and *Rhodotorula* sp. NS-12

**DOI:** 10.1038/s41598-023-31639-z

**Published:** 2023-03-28

**Authors:** Minoo Giyahchi, Hamid Moghimi

**Affiliations:** Department of Microbial Biotechnology, School of Biology, College of Science, University of Tehran, Tehran, 1417864411 Iran

**Keywords:** Biotechnology, Microbiology, Environmental sciences

## Abstract

Polyester-urethanes as the most widely used polyurethanes (PUs) are among the most recalcitrant plastics in natural conditions. Among existing approaches for managing and reducing plastic waste, biodegradation as a promising approach to reduce plastic waste pollution has drawn scientific society's attention in recent years. In this study, two polyester–polyether urethane degrading yeasts were isolated and identified as two new strains of *Exophilia* sp. NS-7 and *Rhodotorula* sp. NS-12. The results showed that *Exophilia* sp. NS-7 is esterase, protease, and urease positive, and *Rhodotorula* sp. NS-12 can produce esterase and urease. Both strains can degrade Impranil^®^ as the sole carbon source with the highest growth rate in 4–6 and 8–12 days, respectively. SEM micrograph revealed PU degradation ability in both strains by showing so many pits and holes in treated films. The Sturm test showed that these two isolates can mineralize PU to CO_2_, and significant decreases in N–H stretching, C–H stretching, C=O stretching, and N–H/C=O bending absorption in the molecular structure of PU were revealed by the FT-IR spectrum. The detection of the deshielding effect in chemical shifts of the H-NMR spectrum after the treatment also confirmed the destructive effects of both strains on PU films.

## Introduction

Plastic is a general term, that refers to vast groups of polymers that are extensively used in different industries and daily life^1^. Despite their low-cost, excellent properties, and easy manufacturing that provide them with this large market share, they are recalcitrant to degradation^2,3^. Besides, poor management of plastic wastes faces the environment and living organisms serious risks. For example, in 2015, it has been reported that only 9% and 12% of global plastic wastes were recycled and incinerated, respectively and the rest of them were landfilled or disposed to nature^3–5^. Polyethylene (PE), polypropylene (PP), polyethylene terephthalate (PET), and polyvinyl chloride (PVC) are classified as thermoplastics and are recyclable, but thermosets are kinds of polymers that cannot be heated and remodeled after the production process^6^. Polyurethanes (PUs), are a diverse group of synthetic heteropolymers, which can be synthesized as thermoplastics, thermosets, elastomers, coatings, adhesives, and sealants^7,8^.

The first PU polymer was produced in 1937 by Otti Bayer^9,10^ and found its way to the industry, a decade later^11^. Nowadays polyurethanes are produced by the reaction of di-isocyanate with a polyol and a chain extender^12^. Depending on the type of polyol, PUs are classified into four groups: polyester, polyether, polycaprolactone, and polyacrylic urethanes. Among them, Polyester polyurethanes are the most widely used^13^, which are more recalcitrant and durable than other types^7^. About 10 million metric tons of polyurethane are produced and used per year in paints, adhesives, manufacturing of tires, fibers, and plastic foams^14^.

PU wastes are mostly landfilled, where due to their structural complexity, low oxygen availability, and high resistance to decomposition, remain as polymers for decades, and a part slowly decomposed, causing the release of pollutants, such as 4,4′-methylenedianiline and 2,4′-toluene diamine^15–17^. In addition, it has been proven that other physical and chemical degradation methods are not sufficient enough to remove PU wastes^17^. Incineration is an example that may reduce the volume of waste but if it is done improperly, can emit toxic gases into the atmosphere^10,18^. Recycling as a waste management method is also complicated and costs a lot as has got a complex process that includes: collection, sorting, size reduction, cleaning, and further separation, requiring various facilities and skills^19^.

In the last few decades, biodegradation has attracted the attention of scientific society as an eco-friendly and cost-effective alternative for polymer waste removal^16,20,21^. There are several studies since the 1960s, reporting potential PU degrading microbial strains and enzymes, reviewed by:^16,22,23^. Most of the reported strains are bacteria and mold fungi, some of them are mentioned as follows: *Serratia* *rubidaea*^24^, *Rhodococcus* *equi*^25^, *Micrococcus* sp.^26^, *Corynebacterium* sp., *Bacillus* *subtilis*^27^, *Bacillus* *subtilis* MZA-75, *Pseudomonas* *aeruginosa* MZA-85^28^, *Acinetobacter* *gerneri* P7^29^, *Comamonas* *acidovorans* TB-35^30^, *Corynebacterium* sp. B12^31^, *Staphylococcus* *epidermidis* KH11^32^, *Alternaria* *dauci*^33^, *Alternaria* *solani*a^34^, *Aspergillus* *niger*^35^*,* *Nectria* *gliocladiodes*^36^*,* *Aspergillus* sp. strain S45^37^, etc. Despite the importance and potential of yeasts in bioremediation and environmental pollution decomposition^38^, a limited number of studies have been conducted on their role in plastic bioremediation, and mostly biodegradable types. Poly ɛ-caprolactone (PCL) film and foam plastic biodegradation by *Pseudozyma* *japonica* sp.^39^ and hydrolytic activity of cutinase-like enzyme from *Cryptococcus* sp. S-2 on polylactic acid^40^ are two of these examples. In 2014, Zafar et al. reported the presence of *Candida* *rugosa* on the surface of the buried polyester PU among the fungal community of commercial compost^41^, but the activity and degradation potential of this yeast on PU film has not been mentioned.

In this study, we isolated and evaluated potential polyester-polyether urethane degrading yeasts from forest soils and wastewater and introduced two strains with the ability to biodegrade PU films.

## Materials and methods

### Materials

Poly [4,4'-methylenebis (phenyl isocyanate)-aH-1,4-butanediol/di (propylene glycol)/polycarbonate] was purchased from Sigma-Aldrich, GmbH, Germany. Impranil^®^ DLN w50 was a gift from Bayer Company. Modified M9 medium was used for Impranil^®^ and PU degradation assays which contains (g L^−1^): Na_2_HPO_4_ (13.0), KH_2_PO_4_ (3.0), NH_4_Cl (1.0), NaCl (0.5), agar (15) and 1% Impranil^®^ DLN w50 (sterilized separately by 0.45 μm filter and was added after autoclaving)^42^ or PU films as the sole carbon source. Urea agar was used for urease activity assay which contains (g L^−1^): peptone (1), NaCl (5), KH_2_PO_4_ (2), phenol red (0.012), glucose (1) and agar (15), Urea (20) in 1 l of distilled water (Urea was sterilized separately by 0.45 μm filter and was added after autoclaving)^43^. Tributyrin Agar (TBA) was used for the esterase activity assay. For TBA preparation, 20 g tributyrin agar base (containing yeast extract and peptone) was added to 1 l of distilled water and 1% (v/v) of tributyrin was added to the base medium before autoclaving and stirred vigorously to emulsify tributyrin properly^44^. All of the above-mentioned mineral salts and media were of analytical grade and purchased from Sigma-Aldrich, GmbH, Germany. Internal transcribed spacers (ITS) amplification was performed using ITS1 (5'-TCCGTAGGTGAACCTGCGG-3') and ITS4 (5'-TCCTCCGCTTATTGATATGC-3') primer sets^45^.

### Sample collection

To isolate polyurethane degrading yeasts, 21 samples were collected from 5 to 10 cm depth of Saravan forest soil (Gilan province, Iran), and wastewater of edible oil from Ladan Oil Company (Tehran province, Iran). Soil and wastewater samples were collected in sterilized zip plastic bags and sterilized glass bottles respectively. Samples were transferred quickly to the laboratory at 4 °C and immediately used for enrichment culture.

### Preparation of polyurethane films

Polyurethane films were prepared by dissolving 0.25 g of Poly [4,4'-methylenebis (phenyl isocyanate)-aH-1,4-butanediol/di (propylene glycol)/polycarbonate], in 25 mL Tetrahydrofuran (THF) solvent. The prepared solution was poured into glass Petri dishes and allowed to evaporate under a fume hood. After 24 h, polyurethane films were cut into quadratic pieces of 2 × 2 cm^2^^5^.

### Isolation of polyurethane degrading yeasts

Environmental yeast strains were isolated via streak plate technique on Rose Bengal Agar (Sigma-Aldrich, Germany). This technique was performed to enrich and isolate yeast strains with the potential of producing polyurethane-degrading enzymes. Further purification was performed on GPY (Glucose-Peptone-Yeast Extract) culture medium until complete purification of all isolates. Purified isolates were stocked in 20% glycerol-containing Tryptic soy broth (TSB) media (Sigma-Aldrich, Germany) and stored at −20 °C.

### Preliminary screening

Protease, urease, esterase, and impralinase activity assays were investigated for screening and selection of isolated strains. To detect the protease activity, isolated strains were cultured on skim milk agar (Merck, Germany)^46^ and esterase activity was evaluated on TBA medium. Inoculated media were incubated at 30 °C for 48 h. Colonies with clear halo zones were considered protease and esterase positive. Slant urea agar was used for urease assay. Prepared culture media was inoculated and incubated at 30 °C for 48 h and color changes were monitored^43^. Impranilase assay was performed using a modified M9 medium with Impranil^®^ as the sole carbon source to screen polyurethane-degrading yeast strains. Culture plates were incubated at 30 °C and monitored frequently for halo zone formation. For quantitative analysis of Impranil^®^ degradation activity, 50 mL of M9 liquid culture media containing 1% Impranil^®^ were inoculated with 5% inoculum with equal turbidity of 0.5 McFarland standard and incubated at 30 °C for 14 days at 120 rpm. In 48 h intervals, 2 mL of the media was recovered from culture for colony counting on GPY agar and Impranil^®^ concentration, measured by spectrophotometer (Shimadzu-UV160, Japan) at 600 nm. The standard curve of Impranil concentration was constructed with different concentrations of Impranil^®^. It is worth mentioning that all experiments were repeated three times and quantitative results were confirmed by t-test analysis (P < 0.05). Statistical analysis was performed by SPSS for windows (version 16).

### Molecular identification of selected yeast strains

Strain identification was performed through PCR amplification of the ITS region of the rRNA gene. DNA was extracted from biomass pellets according to Zhang et al.'s protocol^47^. Amplification was performed by ITS1 and ITS4 primer sets, in a thermocycler with the following program: initial denaturation at 94 °C for 5 min; 30 cycles of denaturation at 94 °C for 30 s, annealing at 57 °C for 30 s, and extension at 72 °C for 1 min, followed by a final elongation step at 72 °C for 10 min. The amplified PCR products were sequenced by Macrogen (South Korea). Sequences were blasted with National Center for Biotechnology Information (NCBI) sequences and the closest identity results were reported and the phylogenetic trees of the identified strains were drawn by the MEGA 7.0.26 application tool.

### Polyurethane degradation assays

To investigate polyurethane degradation, the prepared polyurethane films were added into Erlenmeyer flasks, containing 20 mL of M9 culture medium and each flask was inoculated with 5% of inoculum with equal turbidity of 0.5 McFarland standard. Inoculated flasks were incubated at 30 °C in a shaker incubator at 120 rpm for 30 days. After 30 days, possible changes in polyurethane film structure were investigated by the following tests: Sturm test, Fourier transform infrared (FT-IR) analysis, scanning electron microscopy (SEM), and proton nuclear magnetic resonance (H-NMR) spectroscopy. Inoculum-free medium containing PU films was used as a negative control.

### Carbon dioxide (CO_2_) production assay (Sturm test)

The Sturm test was performed to measure CO_2_ production as an indicator of growth and PU degradation. This test was carried out in silicon-sealed caped vessels with oxygen inlet and CO_2_ outlet. Three pieces (2 × 2 cm^2^) of polyurethane film were added into vessels, containing 50 mL of M9 culture medium. Oxygen gas was blown into the medium through a silicon hose and a 0.45 μm filter to remove the carbon dioxide completely. Then, the outlet and inlet valves were closed and vessels were incubated at 30 °C in a shaker incubator at 120 rpm for 30 days. To measure the CO_2_ production, barium chloride buffer (0.1 M) was added to the KOH buffer (1 M), instill. Then, the CO_2_ outlet was connected to a buffer chamber and white precipitates of barium carbonate were dried by dry heat at 80 °C. The amount of CO_2_ was measured by the difference in the weight of samples dried precipitates and control. This is an indirect way to measure accumulated CO_2_ in the sample, according to this equation: BaCO_3_ → CO_2_ + BaO. The test was repeated three times and Inoculum free M9 medium vessels (containing polyurethane film) were used as control.

### Fourier transform infra-red (FT-IR) analysis

FTIR analysis was performed for chemical bonds and functional group changes elucidation in polyurethane films. For this purpose, after 30 days of incubation, polyurethane films (2 × 2 cm^2^) were recovered from culture media and washed with distilled water. Treated films were placed in a sample plate of FT-IR (Bruker Vector22-Germany) and spectra were recorded within 400–4000 cm^−1^ wavelengths. The intensity was determined based on absorption (%) and polyurethane films incubated in inoculum free M9 medium were used as control.

### Scanning electron microscopy (SEM)

Scanning electron microscopy was performed to evaluate structural changes on the surface of polyurethane films. Polyurethane films were recovered from culture media and fixed with 2% (v/v) glutaraldehyde for 2 h. After fixation, polyurethane films were dehydrated by 25%, 50%, 75%, 90%, and 100% ethanol (2 h for each concentration). Dried polyurethane films were coated with a thin layer of gold and observed by scanning electron microscope *(*EVO18; Zeiss)^48^. The polyurethane films incubated in an inoculum-free M9 medium were used as control.

### Proton nuclear magnetic resonance (H-NMR) spectroscopy

Polyurethane polymer has got many hydrogen atoms in its structure, so H-NMR 500 MHz was used as one of the chemical changes assessments in this experiment. For H-NMR spectroscopy, 30 days treated PU films, were recovered from culture media, washed with distilled water, and dissolved in 0.6 mL of deuterated tetrahydrofuran solvent. After the complete dissolution of polyurethane films in the solvent, the sample was placed in Bruker (Germany) instrument and investigated by H-NMR spectroscope. The polyurethane films, incubated in inoculum-free M9 medium were used as control.

## Results and discussion

### Isolation, purification, and preliminary screening

A total of 15 yeast strains were isolated from 21 samples and screened by Protease, esterase, and urease activity evaluation with the following results: 40% of isolates produced all of the mentioned enzymes, 40% of them produced two and 20% of isolates produced only one of them. As shown in Table 1, isolates No. 3, 7, and 10 have the highest rates of enzymatic activity. According to previous studies, one of the most important factors in PU biodegradation is the potential of related microorganisms to produce certain enzymes such as urease, esterase, and protease, especially in fungal strains which to the best of our knowledge, have not yet been reported to be able to produce more specific enzymes like polyurethanase that was found in certain bacterial strains.

**Table 1:** Enzymatic activity of isolated strains.

Isolate No	Esterase activity	Urease activity	Protease activity
1	++	++	−
2	+++	+++	−
3	+	++	+++
4	+++	++	−
5	++	+++	−
6	−	+++	−
7	+++	+++	+
8	+	−	−
9	+	+++	−
10	++	+++	+
11	+	−	−
12	++	+++	−
13	++	−	−
14	+	−	−
15	++	−	−

− (negative), + (positive). In the case of protease and esterase assays, no halo zone; 1 to 1.5 cm, 1.5 to 2 cm and more than 2 cm halo zone diameters, were reported as−, + , ++ and +++ , respectively. In the case of urease assay, no color change, weak, medium and strong color changes were reported as -, + , ++ and +++ , respectively.

### Impranil^®^ degradation assay

**Figure 1:**
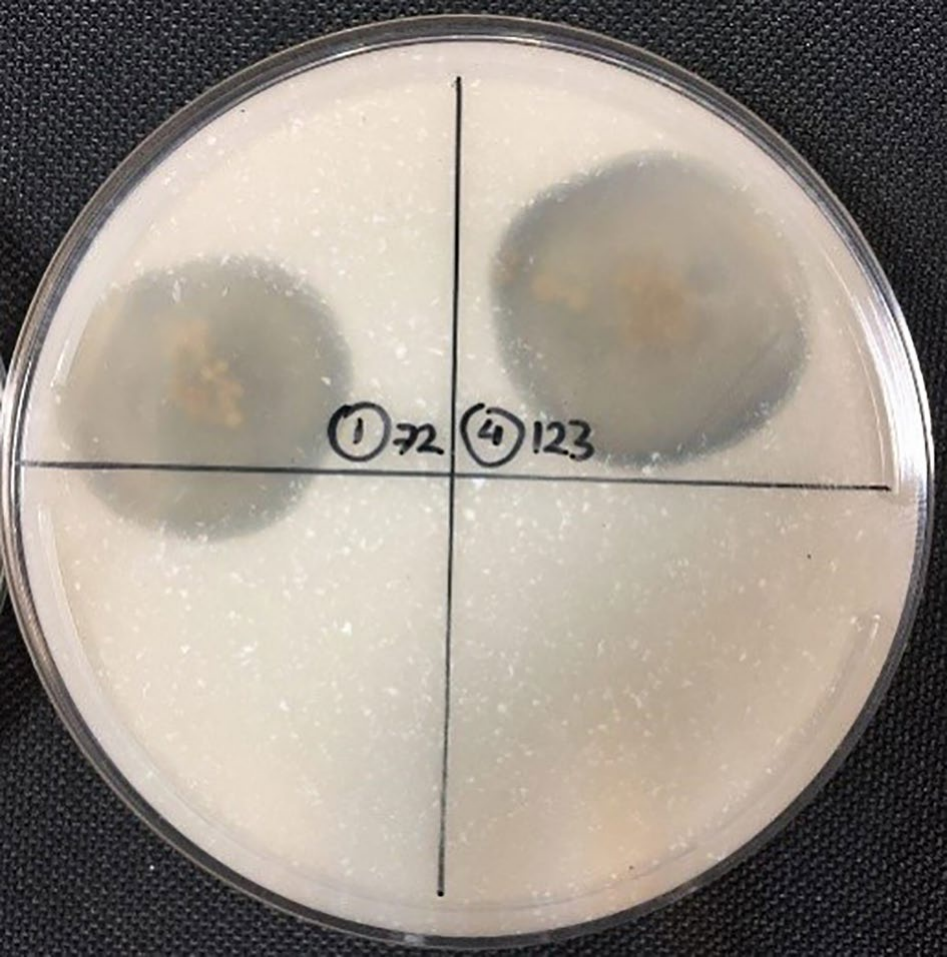
Qualitative analysis of Impranil^®^ degradation. NS-7 (left), NS-12 (right).

According to the results of the qualitative test (Fig. 1), isolates No. 7 (NS-7) and 12 (NS-12) were able to use Impranil^®^ as their carbon source and produced extensive halo zones, in 3.2 and 2.4 cm diameter, respectively.

**Figure 2:**
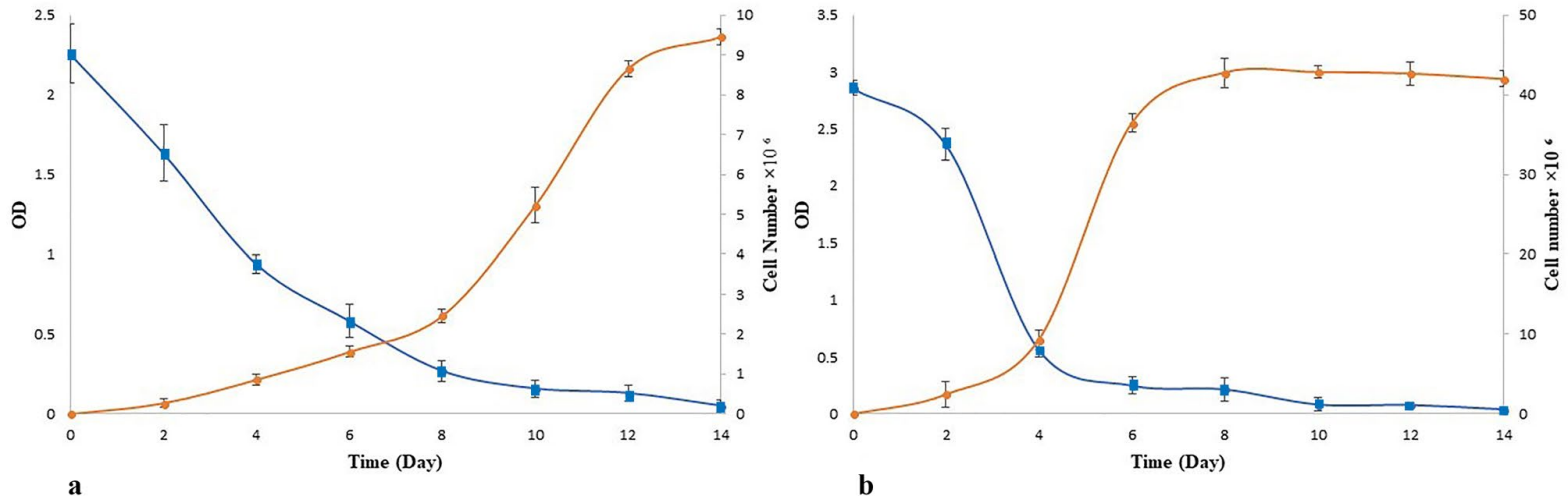
Quantitative results of Impranilase activity (line with blue square) and growth rate (line with orange circle) of selected isolates on Impranil^®^ as the sole source of carbon. (**a,b**) Growth and Impranil^®^ degradation rate of NS-7 and NS-12 in 14 days, respectively**.**

The results of the quantitative analysis showed that the highest growth rate of NS-7 and NS-12 on Impranil^®^ is between (days) 8–12, and 4–6, respectively. Also, the maximum rate of Impranil^®^ degradation of NS-7 and NS-12 was between (days) 0–4 and 2–4, respectively (Fig. 2). Based on the results, NS-12 had the utmost potential for Impranil^®^ degradation. To the best of our knowledge, one of the highest reported potentials of Impranil degradation among fungal strains has been in *Embaria* *clematidis* with 88.84%^8^. Our study showed that at the same duration of time (14 days), both NS-7 and NS-12, also had great performances on this substrate as the sole carbon source, especially in the case of NS-12, with 91.2% degradation.

### Molecular identification of selected isolates

**Figure 3:**
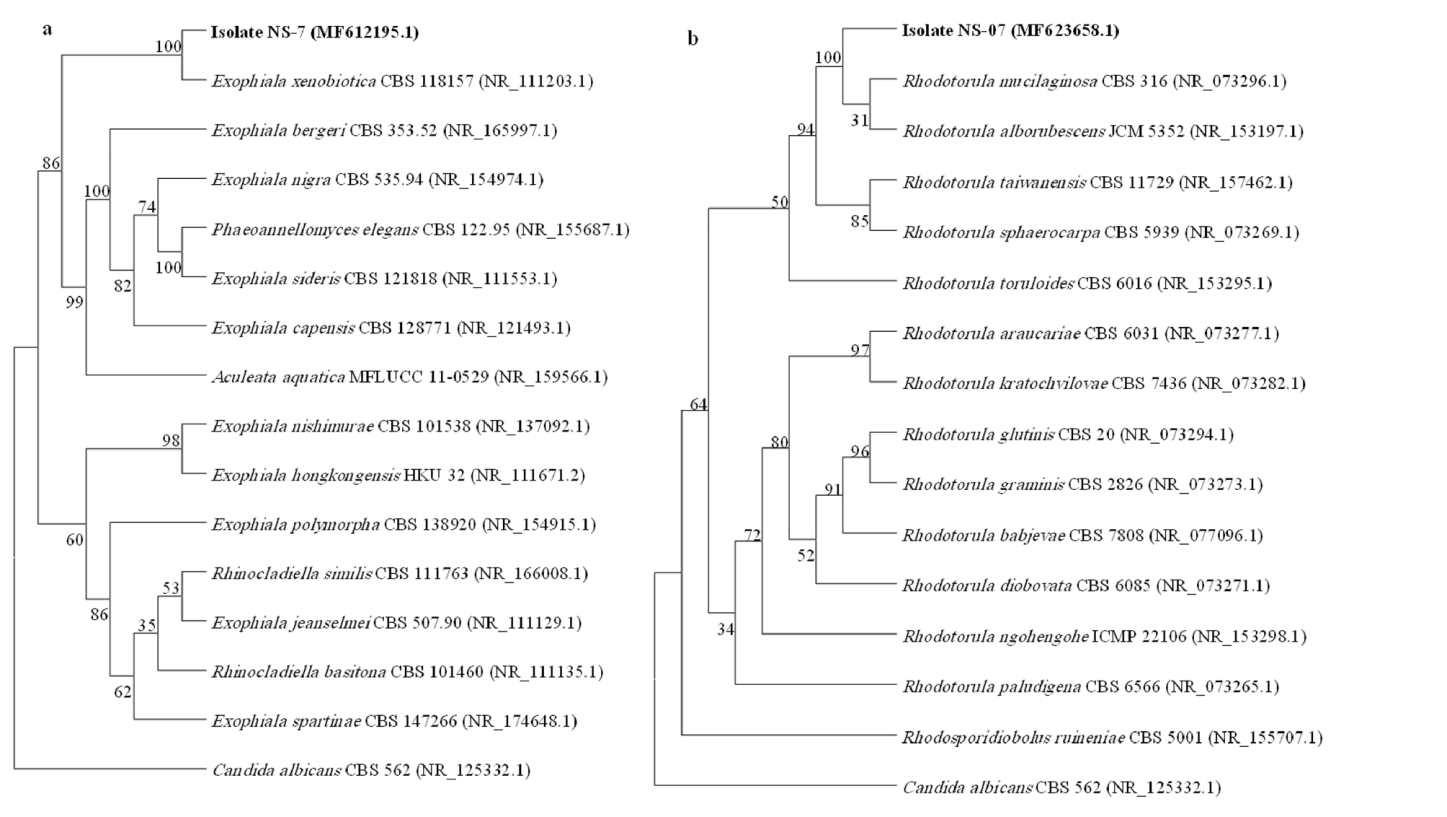
Phylogenetic tree of (**a)** NS-7 with the sum of branch length = 0.99909280 and (**b)** NS-12 with the sum of branch length = 0.89962858 using the Neighbor-Joining method with the bootstrap of 1000. The evolutionary distances were computed using the Maximum Composite Likelihood method and are in the units of the number of base substitutions per site. The analysis involved 16 nucleotide sequences. Codon positions included were 1st + 2nd + 3rd + noncoding. All positions containing gaps and missing data were eliminated. Evolutionary analyses were conducted in MEGA 7.0.26. *Candida* *albicans* CBS 562 was considered as the out-group strain and the accession number of strains is written in front of each taxon.

Two selected isolates (NS-7 and NS-12) were identified by ITS region gene sequencing and compared with retrieved nucleotide sequences on the NCBI server via the BLAST alignment tool. Based on the results, NS-7 was closely related to *Exophilia* *xenobiotica* CBS 118157 with 98.99% ITS sequence identity, and NS-12 shows 99.82% ITS sequence similarity with *Rhodotorula* *mucilaginosa* CBS 316*.* Phylogenetic trees of NS-7 and NS-12 show both in the nearest but distinct sub-branch to aforementioned strains, indicating that these isolates are in the same genus as these hits but their distinction from *Exophilia* *xenobiotica* CBS 118157 and *Rhodotorula* *mucilaginosa* CBS 316 indicates their separation during their evolution from these strains which prove their separate identity (Fig. 3)*.*

The sequence data of *Exophilia* sp. NS-7 and *Rhodotorula* sp. NS-12, are available on the NCBI database under MF612195.1 and MF623658.1 accession numbers, respectively.

### Carbon dioxide (CO_2_) production assay (Sturm test)

The Sturm test was investigated to measure the CO_2_ production rate in selected isolates. The results revealed that NS-12 released 11.76 g L^−1^ CO_2_ in 30 days which was 9.44 g L^−1^ more than evolved CO_2_ by NS-7 with 2.32 g L^−1^. As the release of CO_2_ is an indirect way to evaluate the growth and metabolism rate of aerobic microorganisms, according to the results of this test, we can claim that NS-12 had better growth and degradation potential on PU as the sole carbon source at the same duration.

Also, if we compare the results of the Sturm test in other reports with our study, we can claim that NS-12 grew effectively on PU. For example, Shah et al. examined the quantity of carbon dioxide production by *Bacillus* *subtilis* MZA75 and *Pseudomonas* *aeruginosa* MZA85 on PU which were 7.08 and 6.54 (g L^−1^), respectively^49^.

### Fourier transform infra-red (FT-IR) analysis

**Figure 4:**
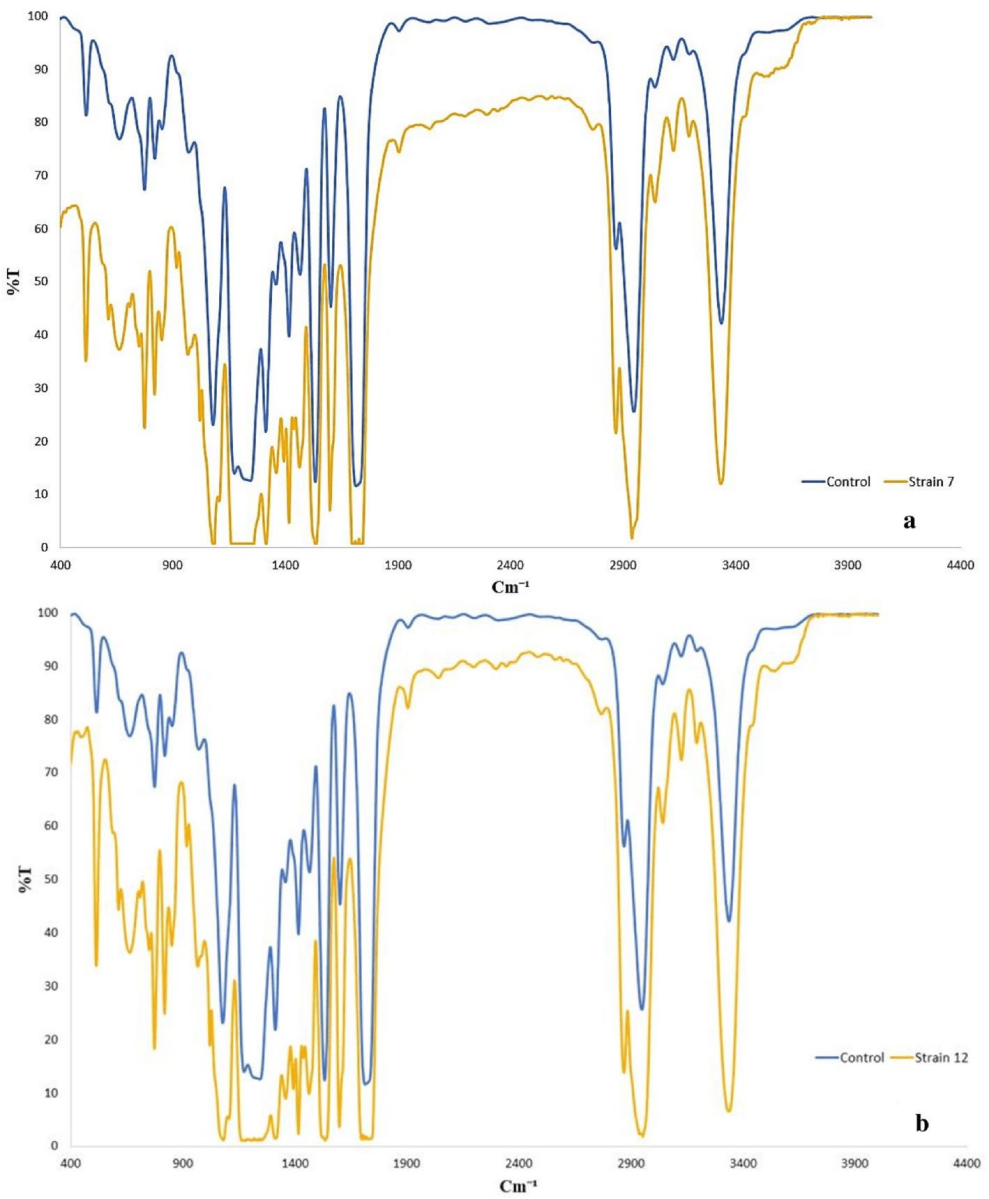
FT-IR spectroscopy of treated polyurethane films and control at 400–4000 cm^−1^ wavelengths. **(a)** Polyurethane film treated by *Exophiala* sp. NS-7. **(b)** Polyurethane film treated by *Rhodotorula* sp. NS-12. In each case, treated polyurethane films were compared with the control.

FT-IR spectrum showed the results as the absorption versus wavelength, to elucidate the changes in functional groups of PU films after treatment. Comparing the spectrum of the control with both of the treated films, showed an obvious shift along the y-axis, indicating the changes in the intensity of NS-7 and NS-12 treated films (Fig. 4). Both isolates showed significant bond breakage ability of PU that can be revealed through the decrease of the absorption value at several peaks, including 3100–3500, 2850–3000, 1700–1750, and 1550–1670 cm^−1^ (Table 2) which indicates that N–H stretching, C–H stretching, C=O stretching, and N–H/C=O bending were affected by treatment with generally better performance by NS-7 strain on degrading this polymer.

**Table 2:** Changing in the absorption of functional groups after treatment.

Functional group	Wave number (cm^−1^) T%^a^	Control	*Exophiala* sp. NS-7	*Rhodotorula* sp*.* NS-12
O–H/N–H	3100–3500	3336 42.2%	3334 12.05%	3334 6.63%
C–H	2850–3000	2946 25.64%	2939 1.75%	2935 2.359%
C=O	1700–1750	1708 11.85%	1693 0.82%	1695 1.394%
N–H/C=O	1550–1670	1600 45.35%	1596 7.069%	1596 3.66%

^a^T%: absorption (%).

### Scanning electron microscopy (SEM)

**Figure 5:**
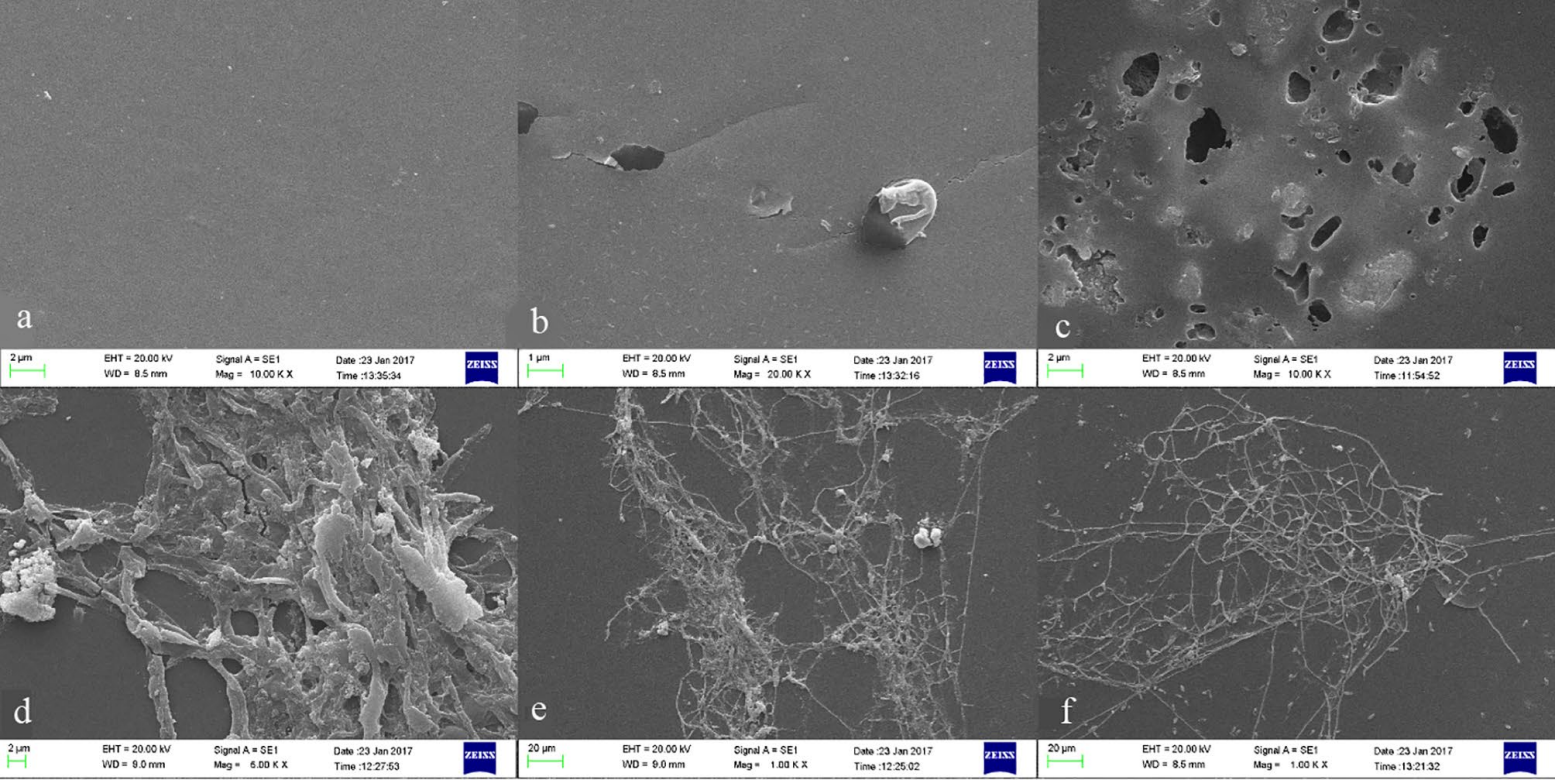
Scanning electron micrographs. **(a)** Polyurethane film without treatment, **(b,c)** holes and pits due to biodegradation of polyurethane films by *Rhodotorula* sp. NS-12 and **(d,e)** biofilm formation on PU film surface by *Rhodotorula* sp. NS-12, **(e)** biofilm formation on PU film by *Exophilia* sp. NS-7.

The surface structure changes of treated polyurethane films were examined by scanning electron microscopy. SEM micrographs showed fungal biofilm formation on the surface of treated polyurethane films and obvious pits and holes were revealing structural changes on the film surface (Fig. 5). As shown in Fig. 5C, several cavities were created that indicated the ability of the strain in polyurethane fragmentation. There are several reports with the same results that assessed the fungal degradation of polyurethane films by numerous pits and holes observed in SEM graphs. Oceguera-Cervantes et al. and shah et al. studies are some of these examples^5,50^.

### Proton nuclear magnetic resonance (H-NMR) spectroscopy

**Figure 6:**
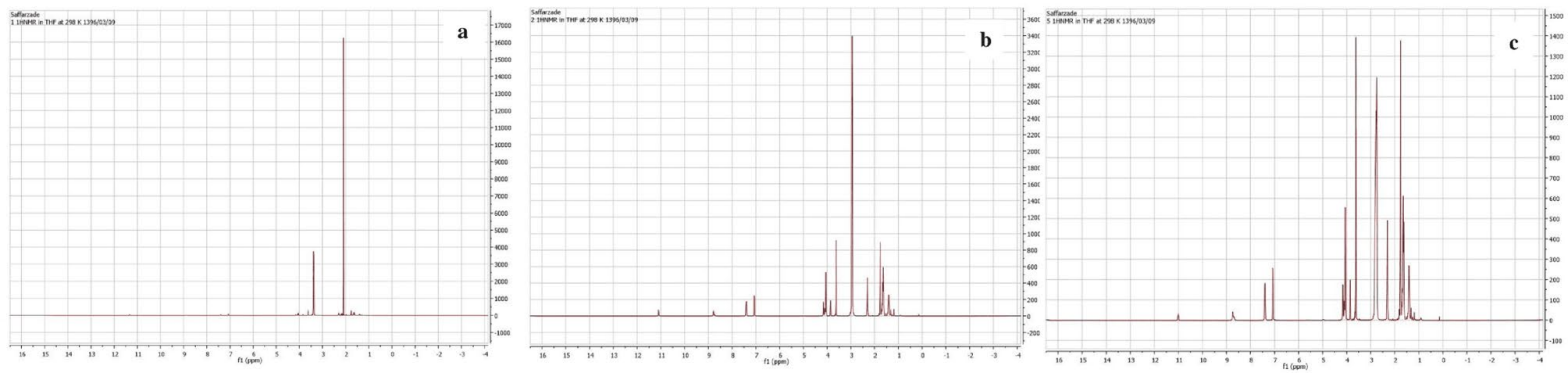
H-NMR spectrums. **(a)** Untreated films, **(b)** treated films by *Exophiala* sp. NS-7, **(c)** treated films by *Rhodoturola* sp. NS-12.

The results of the magnetic resonance spectroscopy of the nucleus are shown in Fig. 6. As can be seen in the NMR spectrum of the treated samples, various new peaks have appeared after 30 days of incubation compared to the control, indicating that both strains could influence polyurethane bonds and chemical structure (Fig. 6)*.* The spectrum of both treated samples, shows the following chemical shifts (δ): a quartet peak at 1–2, two singlet peaks at 2–2.5 and 2.5–3, a quartet peak at 3.5–4 and four singlet peaks at 7–7.5, 8.5–9 and 11 ppm. These chemical shifts can be referred to alkyl or allylic groups, carbonyl, CHα-N, CH_2_-X, aromatic and carboxylic acid groups, respectively^51^. Both treated PU films' spectrum showed a deshielding effect compare to the control with almost the same algorithm. This effect could be the result of the formation of hydrogen bonds, inductive effect and anisotropy in bonds which indicates the changes in chemical structure and formation of new functional groups by both strains. Another important point is the lack of any peak in the chemical shift zone of phenolic groups (4–7 ppm) which their quick and long-term toxic effects on humans and living organisms have been proved. As biodegradation is considered a sustainable and eco-friendly process in xenobiotic removal, this is an important issue should be considered that the degradation process should not leave over more hazardous and toxic compounds than the primitive material in the environment.

## Conclusions

This study introduces two new isolated yeast strains with the potential of degrading polyester-polyether urethanes. Our results show that *Exophilia* sp. NS-7 and *Rhodotorula* sp. NS-12 can successfully degrade PU as the sole source of carbon. FTIR, H-NMR, and SEM micrograph examinations revealed that *Rhodotorula* sp. NS-12 has more destructive and degradative activity on the structure of the PU films and the Sturm test indicates that this strain grows and metabolizes this polymer, better and faster which brings up the possibility that this strain may have an effective and specific enzyme(s), that further studies are needed to find their structure and mechanisms of action on PU. It is worth nothing to mention that this is the first study on the ability of a *Rhodotorula* sp. strain to degrade polyurethanes.

## Data Availability

The data analyzed during this study are included in this manuscript file. DNA sequence data generated for this study are published on the NCBI GenBank online sequence depository under the accession numbers MF612195.1 and MF623658.1.
